# Does the Gut Microbiota Influence Immunity and Inflammation in Multiple Sclerosis Pathophysiology?

**DOI:** 10.1155/2017/7904821

**Published:** 2017-02-20

**Authors:** Monika Adamczyk-Sowa, Aldona Medrek, Paulina Madej, Wirginia Michlicka, Pawel Dobrakowski

**Affiliations:** Department of Neurology in Zabrze, Medical University of Silesia, ul. 3-go Maja 13-15, 41-800 Zabrze, Poland

## Abstract

*Aim.* Evaluation of the impact of gut microflora on the pathophysiology of MS.* Results*. The etiopathogenesis of MS is not fully known. Gut microbiota may be of a great importance in the pathogenesis of MS, since recent findings suggest that substitutions of certain microbial population in the gut can lead to proinflammatory state, which can lead to MS in humans. In contrast, other commensal bacteria and their antigenic products may protect against inflammation within the central nervous system. The type of intestinal flora is affected by antibiotics, stress, or diet. The effects on MS through the intestinal microflora can also be achieved by antibiotic therapy and* Lactobacillus*. EAE, as an animal model of MS, indicates a strong influence of the gut microbiota on the immune system and shows that disturbances in gut physiology may contribute to the development of MS.* Conclusions.* The relationship between the central nervous system, the immune system, and the gut microbiota relates to the influence of microorganisms in the development of MS. A possible interaction between gut microbiota and the immune system can be perceived through regulation by the endocannabinoid system. It may offer an opportunity to understand the interaction comprised in the gut-immune-brain axis.

## 1. Introduction

### 1.1. Definition of Multiple Sclerosis

Multiple sclerosis (MS) is a progressive disease of the central nervous system (CNS) characterized by the presence of lesions in the brain and the spinal cord [1]. Nylander and Hafler [2] demonstrated that the inflammatory factor in MS contains CD4 and CD8 T cells, B cells, and activated monocytes that result in the degradation of the myelin sheath surrounding nerves. Traditionally, inflammatory demyelination has been considered the primary form of the pathogenesis in MS.

Although the etiology of MS remains unclear, several hypotheses suggest that autoimmunity plays a major role in the development of the disease. The most widely supported view is that MS is a CD4+ T cell-driven autoimmune disorder [3]. In MS lesions, astrocytes play a paradoxical role during disease development [4]. Experimental data show that astrocytes not only mediate inflammation but also diminish the detrimental effects of proinflammatory factors. Activated astrocytes secrete compounds including reactive oxygen and nitrogen species [5], which have toxic effects on neurons. Oxidative stress is a key factor in the pathogenesis of MS. Activated macrophages and microglia in the CNS produce reactive oxygen species (ROS) and reactive nitrogen species (RNS) and secrete cytokines (tumor necrosis factor (TNF), interleukin-1 (IL-1), and interleukin-6 (IL-6)) and chemokines (macrophage inflammatory protein (MIP-1), monocyte chemoattractant protein (MCP-1), and interferon-gamma- (IFN-*γ*-) induced protein (IP-10)). Neurodegeneration during MS may result from chronic oxidative stress and excitotoxicity [6].

Recent clinical and experimental studies indicate that MS arises from a negative interaction between genetics and environmental factors [7, 8]. Multiple sclerosis has a complex genetic component including the haplotype HLA-DR15 and over 100 of other types of minor risk alleles involved in the immune response. Additionally, several environmental and lifestyle factors contribute to the onset of MS, including latitude, deficiency of vitamin D3, obesity early in life, cigarette smoking, and the Epstein-Barr virus (EBV). Hedström et al. [9] and Alotaibi attempted to find a relationship between the active form of vitamin D3, 1,25-OH D3, and the major histocompatibility complex (MHC) HLA-DRB1*∗*15 allele, which is linked to an increased risk of developing MS [10]. The study results of Hedström and Alotaibi [11] suggest that HLA-DRB1*∗*15 only contributes to the risk of disease when susceptible individuals are deficient in vitamin D.

In the 1970s, a relationship between the 6p21 region of the* HLA* gene and MS risk was discovered. In the following three decades, this region was only considered a genetic risk factor that increased susceptibility to MS. It was not until the introduction of genome-wide association studies (GWAS) that new genetic risk factors were found (the International Multiple Sclerosis Genetics Consortium). There is considerable variability in* HLA *genes in patients with MS. Research in Europe and the United States has revealed the existence of three HLAs that occur more frequently in MS compared to the general population. Further research in the US has shown that MS is more likely to occur in individuals who possess more than one of these HLAs. It can be assumed that the HLA system correlates with the course of the disease [11]. There are at least two genes, apart from HLA-DRA, on which single-nucleotide polymorphisms (SNPs) can occur, which may increase susceptibility to MS: the IL2R gene (an encoding subunit of the IL-2) and IL7RA gene (a subunit of IL-7) (the International Multiple Sclerosis Genetics Consortium). Furthermore, the risk of MS may be altered by polymorphic variants of ALCAM, CD6, CD80, CD86, and CD40 genes. Lastly, there may be sex-related genetic factors that contribute to the risk of MS. Reduced levels of CD6 mRNA in men and a reduced expression of the CD40L gene in women may increase the risk of developing MS (Pender).

Multiple human epidemiological studies have revealed the effects of environmental factors on the prevalence of MS [11] and demonstrated that viral infections, lack of sun exposure, vitamin D deficiency, active or passive smoking, season of birth, obesity, vitamin A deficiency, dietary habits (especially high levels of salt and fat), stress, and the intestinal microflora play a significant role in the initiation of the disease. EBV is a widely recognized risk factor. A seroepidemiological study of MS found that nearly 100% of the patients were infected with EBV [13]. If EBV infection occurs in late childhood, it is considered the largest risk factor for the development of MS. There is a strong EBV-specific CD8+ response in the blood during the onset of MS; the intensity of the response decreases during the course of the illness [14, 15].

Neuropathological studies support roles for the human herpes virus (HHV-6) and human endogenous retroviruses (HTLV-1/2, HIV-1/2) as risk factors for the development of MS. Herpes viruses can reactivate or cause de novo infections, leading to neurological disabilities [16]. Other risk factors include lack of sun exposure and deficiency of vitamin D [17]. Spelman et al. [18] demonstrated latitude-dependent lag between seasonal ultraviolet radiation (UVR) and relapse probability, which showed that the probability of a relapse increases in MS patients living at latitudes further from the equator. Additionally, MS patients living at further latitudes have significantly lower levels of vitamin D in all seasons of the year. These patients reach a low threshold level of 25(OH)D, long immunomodulatory effect sooner after the winter solstice compared to people residing at more equatorial latitudes. Low levels of vitamin D have been observed in other neurological diseases, including Alzheimer's disease, Parkinson's disease, and stroke [19]. A meta-analysis showed that individuals born in autumn have a high risk of MS, based on their exposure to UV light and vitamin D levels [20]. Studies show that the risk of the disease decreases with diets low in salt and fat and with the use of essential oils from* Pterodon emarginatus*, a Brazilian legume [21].

Symptoms of MS include cognitive deficits, impaired information processing, and long-term memory deficits [22, 23]. Clinical symptoms are muscle weakness, blurred vision, dizziness, and fatigue [2].

## 2. Main Text

### 2.1. The Definition of Gut Flora

Gut microflora is the population of microorganisms living in the human gut. Microflora can be loosely divided into harmless, beneficial, or pathogenic forms. Intestinal microflora develops within the first few years of life and then remains largely stable. There are an estimated 1000+ different types of intestinal flora (Table 1). The main factors that influence the composition of the intestinal flora after birth are the duration of pregnancy and the method of delivery (natural childbirth or caesarean section) [24]. Premature birth is associated with the presence of* Staphylococcus*. Children born vaginally have a higher incidence of* Lactobacillus* and* Prevotella,* while children born by caesarean section have a higher incidence of* Staphylococcus*,* Corynebacterium*, and* Propionibacterium* [25]. Facultative anaerobic bacteria such as* Escherichia coli* and other coliform bacteria are the first colonizers of the intestine in infants. In the first year of life, the intestine is colonized by* Bacteroides*,* Clostridium*,* Ruminococcus*, and* Bifidobacteria* [26]. Breast-feeding promotes* Bifidobacteria* and* Lactobacillus *proliferation. The intestinal microflora of a 3-year-old child is identical to that of an adult [27, 28]. Mariat et al. [29] suggested that the changes in the composition of the intestinal flora might result in the dysfunction of the immune system in elderly adults. Collins et al. [30] reported that the adult intestinal flora is mainly composed of four main clusters:* Firmicutes*,* Bacteroidetes*, Actinobacteria, and* Proteobacteria*. Tuohy et al. [31] reported that the intestinal flora can be divided into three groups, including health benefits, bacteria of the genera* Bifidobacterium* and* Lactobacillus*, and opportunistic microorganisms [32, 33].

### 2.2. GALT System

The gut-associated lymphoid tissue (GALT) is the largest immune system in the body. Intestinal bacteria act through the lymphatic system, which is associated with the digestive tract, that is, the GALT system. The GALT consists of organized cellular complexes (Peyer's patches and solitary lymphoid follicles) and dispersed cells (T and B cells, macrophages, and dendritic cells) which are located in the intestinal lamina propria, near the epithelium. In the intestinal epithelium, covering Peyer's patches, are specialized cells, known as M cells, which capture antigens and transmit them to T and B cells. Unlike pathogens, commensal bacteria are unable to penetrate other internal organs or the bloodstream. Weng and Walker reported that antigen stimulation of lymphocytes causes proliferation of naive T and B cells, which are activated in the digestive tract, migrate to the lymph vessels and mesenteric lymph nodes, and enter the bloodstream. In the blood, T and B cells are transported back into the lymphatic structures of the digestive tract and the mucous membranes of other systems (respiratory, urogenital, and endocrine glands), where they remain as effector cells. Germ-free animals had only free B cell. Intestinal microflora colonization by commensal bacteria in GF animals stimulates the immune system, resulting in the formation of active Peyer's patches, proliferation of lymphocytes in the lamina propria, and increased production and circulation of secretory antibodies, mainly immunoglobulin A (IgA) and immunoglobulin M (IgM). Nonpathogenic intestinal bacteria stimulate the formation of primarily natural antibodies, which are essential components of the nonspecific immune mechanisms and constitute the first line of defense in the immune response [34]. Research in the Institute of Mother and Child in Prague on probiotic strains of* E. coli* (O86 and Nissle 1917) showed that neonatal* E. coli* leads to long-term stimulation and production of secretory antibodies. The most commonly used probiotics are strains of* E. coli* Nissle 1917,* Saccharomyces boulardii*, and a probiotic mixture containing four strains of* Lactobacillus* and* Bifidobacterium* and one of three strains of* Streptococcus salivarius* [35]. Probiotics may also directly influence the permeability of the intestinal barrier. In the recent studies on epithelial cell lines derived from the colon tissue, it was confirmed that the probiotic* Lactobacillus acidophilus* restores proinflammatory cytokines such as TNF-*α* and interferon-gamma (IFN-*γ*) [36]. Probiotics contribute to the balance of cytokines and can favorably affect the course of those allergic and inflammatory diseases. The clinical effects of probiotics are beneficial and activities of the specific strain cannot be transferred to another strain of the same species. Clinical observations indicate that the probiotic strains may be potentially useful in the treatment of inflammatory diseases [37].

### 2.3. The Impact of Drugs on the Flora

An increased amount of* Bifidobacterium* was detected in stool samples of people who consume caffeine [38]. The researchers found that smoking and drinking coffee can alter the composition of the intestinal flora. Caffeine in coffee increases the level of granulocyte colony-stimulating (G-CSF) levels, which leads to significant improvement in memory in mice [39].

### 2.4. Interaction of Intestinal Flora with Other Systems

Organisms perform a number of metabolic processes, including the synthesis of vitamins B2, B7, and C, which can affect the bioavailability and metabolism of drugs. Some species of bacteria activate the immune system and can cause the development of inflammatory bowel disease (IBD) and other diseases including myasthenia gravis and diabetes [40]. Turnbaugh et al. [41] demonstrated that the intestinal microflora is related to obesity. In the experiment, human intestinal microflora was transferred to GF mouse and was monitored during manipulation of the diet of mice. The introduction of diet resulted in changes after one day [42]. Increasing the energy production by methanogenic bacteria may contribute to the development of obesity. After surgical treatment of obesity, the number of* F. prausnitzii* in patients with type 2 diabetes (T2D) increased but was lower than that in the controls. After surgery, reduced blood glucose, insulin, and glycosylated hemoglobin were noted in patients and there was also decreased resistance to insulin, based on the ELISA results of HOMA-IR (Homeostasis Model Assessment of Insulin Resistance). Some bacteria, such as Firmicutes, contribute to an increase in the absorption of short-chain fatty acids [43]. The effect of lipopolysaccharides and peptidoglycans on the circulatory system by the permeability of the intestinal epithelial barrier stimulates the production of cytokines. These substances have an impact on the synthesis of low-density lipoproteins and can cause damage to the endothelial cells, foam formation, and proliferation of smooth muscle cells [44], the factors that are closely related to the development of atherosclerosis. In patients with heart failure, colorectal microvascular changes may induce the production of cytokines, which contribute to the impaired myocardial function. The bacteria will also be found in the blood circulation, so they may also play a role in the development of heart failure [45]. Sun et al. [46] demonstrated that cathelicidin antimicrobial peptide that is produced in the beta cells of the pancreas in mice with diabetes is also present in normal mice. In another study, intestinal bacteria were transferred from normal mice into the intestine of mice with type 1 diabetes. The bacteria are involved in the initiation of fatty acid production, which leads to the formation of cathelicidin and inhibiting the development of type 1 diabetes [47]. The immune system protects against the invasion of pathogens on the mucosal surface through the production of IgA. [48]. Germ-free animals reduced the number of intestinal intraepithelial T lymphocytes. T cells are important for the detection of pathogens and antigen presentation. Intestinal colonization by microorganisms is required for optimal growth, development, and function of the intestinal immune system [49, 50]. Microbial action on the central nervous system is expressed by modulating cytokine levels. This action also includes changes in the expression of receptors in the brain which affect the interaction of the intestines and the autonomic nervous system, including regulation of the hypothalamic-pituitary-adrenal axis [51].

### 2.5. The Influence of Gut Flora on the Development and Maturation of the CNS

Changes in the microbiome intestine can lead to abnormal immune responses in both the distal regions of the gut and immune system, that is, the CNS. The microflora modulates the immune system through by-products (Table 2) [52]. Human intestinal flora can play very important role in the pathogenesis of MS, and recent studies suggest that replacing some of the bacterial population in the gut can lead to a state of the proinflammatory potential mechanism of pathogenesis of MS. Bacteria and their antigenic products may protect against inflammation within the central nervous system of man. Specific bacterial antigens, such as polysaccharides (PSA) from* B. fragilis*, mediate the migration of microflora, which indicates a significant interaction between the intestinal mucosa and human brain. These observations suggest that populations of effector cells and regulatory T (Treg) cells are involved in the pathogenesis of MS [53].

Pantazou et al. [54] described the gut-brain axis and demonstrated that intestinal bacteria affect neuroendocrine function and the maturation of the brain by stimulating migration of CD4+ T cells that express CD39 in the brain [55]. Experimental autoimmune encephalomyelitis (EAE) has been used as a model to explore MS and other demyelinating autoimmune diseases of the CNS. Predominantly Th1 and Th17 characterize the immune response to EAE [56]. Lee et al. have shown that oligodendrocyte glycoprotein sensitized mice are susceptible to EAE, but the authors concluded that the segmented bacterial colonization caused her susceptibility [57]. EAE resistant mice were also infected with* Lactobacillus casei* Shirota [58] and* Bifidobacterium animalis, *which were found to reduce symptoms [59]. A potential therapeutic strategy for MS is oral administration of probiotic* Lactobacillus* species, which has been shown to result in IL-10-dependent activation of Tregs in the CNS followed by reduction of IFN-*γ*, TNF-*α*, and IL-17. Treatment targeting the gut of EAE mice can suppress chronic inflammation. Effect of intestinal microflora may act to both increase and decrease the occurrence of autoimmune diseases by affecting the permeability of the intestinal immune tolerance to the loss of constituents of the microflora and the formation of antibodies to the antigens of intestinal bacteria [60].

### 2.6. Latitude and Specific Gut Flora

According to Miyake et al. [61], the composition of intestinal microflora in Japanese patients with MS is not significantly different from that of healthy people. However, the analysis of this study by UniFrac showed significant differences (*p* < 0.05) in the overall structure of the intestinal microflora in MS patients and healthy controls. The intestinal microflora in MS patients has greater interindividual variability than that of healthy controls [62–64]. This study also showed a decrease in the percentage of several* Bacteroides*, including* B. stercoris*,* B. coprocola, *and* B. coprophilus* in the intestinal microflora in patients with MS [61].

### 2.7. Effect of Flora on Cytokines in MS

An imbalance of intestinal bacteria, intestinal epithelial cells, and cells of the immune system in the intestinal mucosa can lead to overstimulation of the immune system. Suppression of excessive immune stimulation is controlled by Treg cells, a distinct population of CD4+ T cells produced in the thymus and peripheral organs of the immune system (e.g., GALT). Tregs have inhibitory effects on inflammatory cell populations and autoreactive effector cells. Treg function defects occur frequently in autoimmune diseases such as rheumatoid arthritis, systemic lupus erythematosus (SLE), and MS [65].

According to Berer and Krishnamoorthy [66], there is increasing evidence from animal studies of a relationship between the type of gut microflora and the progression of MS. Autoimmune reactions can be produced by molecular mimicry or by stimulating the production of lymphocytes. Metabolites produced by these bacteria can also affect the autoimmune system. Multiple sclerosis is a demyelinating disease, which coincides with inflammatory processes [67]. Whether bacterial pathogens act as initiators of MS is not entirely clear. The intestinal microflora can produce various metabolic by-products that affect the CNS, causing reduced autoimmunity. These by-products include short-chain fatty acids (acetic acid, butyric acid, and propionic acid) which bind to the anti-binding G protein coupled receptor (GPCR) and produce Treg, which suppresses proinflammatory cytokines and antigen presenting cells. Adenosine triphosphate stimulates the production of Th17 inflammatory cells, which can cause autoimmune reactions in the CNS [68]. This can adversely affect the commensal bacteria* Bacteroides fragilis* and cause T cell differentiation to Th1, IL-10, and Treg [69–71].

Farrokhi et al. [72] demonstrated unique lipodipeptide bacteria which originate from serine lipodipeptide, lipid 654, which is produced by some Bacteroidetes commensal species, and act as ligand-receptor, providing further evidence for an association between the bacteria and the MS. Kleinewietfeld et al. [73] demonstrated that lipid 654 is expressed at much lower levels in the serum of MS patients than in healthy controls. Lipid 654 may be biomarker of MS [72]. Furthermore, these bacteria produce a number of metabolic products known as the metabolome of by-products which could cause activation of the immune system.

### 2.8. Effect of the Flora on MS Symptoms

The earliest noticeable changes in the permeability of the blood-brain barrier are oligodendrocyte apoptosis and microglial activation [74]. At that time, demyelination is still visible [75–77]. Soluble toxins may be initiating factors in MS. Natural toxin of* Clostridium perfringens* or toxins B and D influence neurological symptoms [78–82]. The toxins are absorbed by the intestine, enter the bloodstream, and cause MS-like symptoms (e.g., blurred vision, lack of coordination, or spastic paralysis). Murrell et al. [83] first suggested that exotoxins could be a potential cause of MS, as humans are not natural hosts of* C. perfringens* types B or D [84–87]. Exotoxins bind to receptors present in the vascular system of the brain and in both myelinated and unmyelinated brain regions, such as the corpus callosum [88–91]. Osmolysis occurs upon binding to the receptor [92–96]. Dawson [97] first described the morphology of relapsing multiple sclerosis. Binding of retinal vein which forms a barrier to the CNS may explain the occurrence of periphlebitis retinae in MS patients [98, 99]. Toxins affect the barrier veins, which may cause inflammation of the retina. Periventricular accumulation of monocytes is often observed [77]. During the test, the effect of intestinal microflora has been shown that the colonized mice had more symptoms become severe and inflammation [100]. This was demonstrated in another animal model of MS [101]. Dendritic cells (DCs) may not activate T cells to an autoantigen as effectively as DCs in colonized animals. Commensal bacteria can act as either the preferred symbiont or a disease-promoting pathobiont, depending on the target of the autoimmune disease, different effector mechanisms, and the composition of the intestinal flora [102].

### 2.9. Effect of Immunomodulatory Agents in MS on the Gut Flora

Further evidence of a link between bacteria in the gut and MS is the decreased concentration of* Faecalibacterium* in patients with MS compared with healthy subjects [103]. Butyrate production is associated with increased numbers of Treg cells, which suggests a potential mechanism through which changes in the intestinal microbiome may lead to predisposition for developing MS. Patients with MS treated with glatiramer acetate have less Bacteroidaceae,* Faecalibacterium*,* Ruminococcus*, Lactobacillaceae,* Clostridium*, and other members of the Clostridiales class compared with untreated MS patients. The study compared 3 groups: healthy subjects, patients with MS who are treated, and patients who are not treated. In response to vitamin D supplementation, only MS patients who did* not* undergo glatiramer acetate treatment showed an increase in the number of* Akkermansia*,* Faecalibacterium,* and* Coprococcus*. It was recently discovered that intestinal colonization by the* Clostridium perfringens* type B is associated with relapse in MS. Toxins produced by* C. perfringens* can lead to microvascular complications leading to neuronal and oligodendrocyte damage [104–107], which may serve as a trigger for future demyelinating events in susceptible individuals. Jhangi et al. [108] compared MS patients with healthy subjects and observed increased concentrations of Archaea (*Methanobrevibacter*) and decreased concentrations of* Butyricimonas* and Lachnospiraceae in MS patients.

According to Wekerle [8], MS also arises from a negative interaction between genetics and environmental factors. A large number of genes promote an autoimmune response against brain cells. New experimental and clinical studies indicate that autoimmune attacks are triggered by an interaction between immune cells of the brain and the gut microbiota. These findings may contribute to the development of new therapeutics that modulate gut microbiota.

Cannabinoid receptors in the intestinal tissue are activated by ligands of cannabinoids that may activate cannabinoid receptors in other local systems. Chiurchiù et al. [109] discovered that treatment of MS patients with anandamide affects the cannabinoid receptors and leads to a decrease in TNF-*α* and IL-6 production. Recent discoveries have shown that specific microorganisms of the intestinal microflora can improve the clinical symptoms of EAE; that is, strains of lactic acid bacteria can enhance the immunological activity of B cells and Treg by increased production of IL-10.

According to Miller et al. [110], MS is an autoimmune disease of the brain, because antagonists of TNF-l improve the condition of many patients with autoimmune disease (e.g., Crohn's disease, ankylosing spondylitis, and rheumatoid arthritis). TNFR2 deficiency causes spontaneous autoimmune-driven demyelination in the brains of transgenic female mice and increases the levels of IL-17, IFN-*γ*, and IgG2b. This is due to the composition of intestinal microflora, as autoimmunity persists after oral antibiotics. Another result of this study is the impact of the type of intestinal microflora (*Akkermansia muciniphila, Sutterella stercoricanis, Oscillospira, Bacteroides acidifaciens*, and* Anaeroplasma*) in male mice in the Alpine resort, where the microflora resulted in a protective effect.

However, the microflora of female mice mainly consisted of* Bacteroides *sp.*, Bacteroides uniformis,* and* Parabacteroides, *which influenced the brain's autoimmune system. Commensal gut bacteria affect TNFR2 levels, which can cause autoimmune-driven demyelination in mice. Therefore, microflora can contribute to the autoimmune response of TNF in autoimmune diseases of the brain.

Messaoudi et al.[111] reported that EAE CD4+ cells directed against self-antigens pass into the CNS and cause demyelination. Many animals were treated with antibiotic therapy in order to reduce the intestinal microbiota. Antibiotics change the amount and composition of the intestinal flora and can reduce the clinical symptoms of autoimmune diseases of the brain. In the EAE model, broad-spectrum antibiotics reduced the susceptibility to EAE by altering the population of T cells in the GALT and peripheral lymphoid tissues. The growth of lymphocytes provided a protective role against EAE. CD5+ and CD19+ in the distant lymphoid organs decrease the levels of IFN-*γ*, MIP-1a, MIP-1b, MCP-1, IL-17, and IL-6 and increase IL-10 and IL-13 [112]. Small changes in Th1 and Th17 lead to an anti-inflammatory Th2 response. Th1 and Th17 contribute to MS progression, while FOXP3+ Treg cells play a protective role. These results indicate that the change in the composition of the intestinal flora due to antibiotic therapy may be beneficial in the treatment of MS [113]. Segmented filamentous bacteria are related to the introduction of Th17 and have been shown to influence autoimmune diseases [114].

### 2.10. The Effect of Diet on Gut Flora

Diet plays an important role in shaping the gut microbiome. In adults, a change in diet can produce a change in the intestinal microflora. One study found a plant-based diet, which leads to an increase in the population of Firmicutes (*Roseburia*,* Ruminococcus bromii*, and* Eubacterium rectale*) and the transition to a meat-based diet leads to an increase in the number of* Alistipes*,* Bilophila,* and* Bacteroides* [115]. Diet may influence the course of MS. Intestinal microbiota can modulate the effects of autoimmune diseases in humans [57, 116]. Th17 cells were shown to induce divided filamentous bacteria, which play an important role in the pathogenesis of autoimmune disease [117–119]. Some metabolites of commensal bacteria introduce FOXP3 positive Treg cells into the colon [120]. The gut has its own mechanism for the elimination of proinflammatory Th17 cells [12]. Studies have shown that consumption of* Candida* could be a new therapeutic strategy for the autoimmune system. This study suggested that dietary yeast might be an important treatment for autoimmune diseases. Pathological examination showed that the amount of MNC (mononuclear cells) in the spinal cord of mice was lower than those in the control group, which was not treated.

Riccio and Rossano [121] reported that diet can influence the severity of MS in both relapsing-remitting MS and primary progressive MS. Diet can have an impact on symptoms of MS by controlling metabolic processes, inflammatory cells, and the composition of commensal intestinal microflora. Increased permeability of the intestinal barrier can lead to cross-reactivity between proteins, leading to the production of IgG and IgA. A diet high in salt, animal fat, red meat, sugar-sweetened beverages, carbohydrates, and fiber, in addition to a lack of physical activity, may increase symptoms, as these all influence metabolism and can lead to dysbiotic intestinal microflora, low-grade systemic inflammation, and increased permeability of the intestinal barrier. A low calorie diet of vegetables, fruits, legumes, fish, prebiotics, and probiotics can reduce symptoms by maintaining a healthy intestinal microflora symbiosis. These results indicate that microflora changes may cause activation of proinflammatory agents and may contribute to relapse in MS [122].

Burkitt [123] first described the potential protective effect of diet on bowel disease.

While working in Africa in 1960, he noticed a remarkable lack of noncommunicable diseases in the native Africans who consumed the traditional diet, which was rich in fiber. Antimicrobial peptides such as alpha-defensins, which are released by Paneth cells, may also have a significant effect on endogenous bacterial flora.

The reduction in the amount of microorganisms in the intestine is most likely to be an adverse effect of globalization, which leads to the consumption of generic, nutrient-rich, uncontaminated food. Both in the Western world and in developing countries, a diet high in fat, protein, and sugar and low in absorbable fibers is associated with a rapid increase in the incidence of noninfectious intestinal diseases [124].

The transition from a diet rich in fiber and low in fat to a high-fat diet alters the composition of the bacterial flora as early as after day 1 [41]. The intestinal flora of children from nonurban areas of Africa is rich in species, including* Prevotella *and* Xylanibacter*, which break abundant fiber. The resulting distribution of short-chain fatty acids in the flora has immunomodulating properties, which may explain the lower incidence of autoimmune diseases and asthma in these environments [125]. In contrast, animals given “Western” diets have high energy, due to the high sugar and fat content, lower levels of* Bacteroidetes*, and increased levels of Actinobacteria [41].

The relationship between the CNS, immune system, and gut microflora is associated with the development of MS. Possible interactions between the intestinal microflora and immune system can be examined by altering the endocannabinoid system to understand the interactions in the brain-gut axis [126].

## 3. Summary

The etiopathogenesis of MS is not fully known. Studies suggest that risk factors include viral infections, vitamin D deficiency, and smoking. There is also evidence to suggest that gut microflora could be important in the pathogenesis of MS. Recent studies have shown that replacing some of the bacterial population in the gut can lead to a proinflammatory state, indicating a potential mechanism causing MS in humans [53]. Furthermore, the intestinal microflora in MS patients has greater interindividual variability than that of healthy controls [62–64]. In contrast to other commensal bacteria, their antigenic products may protect against inflammation within the CNS. These results indicate that the intestinal microflora in patients with MS is characterized by moderate dysbiosis. Miyake et al. in their study also showed a decrease in the percentage of several* Bacteroides*, including* Bacteroides stercoris*,* Bacteroides coprocola*, and* Bacteroides coprophilus* in the intestinal microflora in patients with MS [61]. The results suggest a negative correlation between* Prevotella copri* and the pathogenesis of MS [61]. According to Ochoa-Repáraz et al., intestinal flora may affect the pathogenesis of MS, as changes in the gut microbiome lead to an irregular immune response, in both the colon and distal regions of the immune system, that is, the CNS. Specific bacterial antigens, such as PSA from* Bacteroides fragilis*, mediate the migration of the CNS, indicating a substantial interaction between the intestinal mucosa and the brain [69]. These observations suggest that the population of effector cells and regulatory cells involved in the pathogenesis of MS in humans may be associated with the lymphoid tissue of the gut [53]. Patients with MS had reduced concentrations of* Faecalibacterium* [103]. Furthermore, MS patients treated with glatiramer acetate showed an increase in Bacteroidaceae,* Faecalibacterium*,* Ruminococcus*, Lactobacillaceae,* Clostridium*, and other members of the class Clostridiales when compared with untreated MS patients.

## Figures and Tables

**Table 1:** The composition of human intestinal flora.

The name of the bacteria	The amount of bacteria	Age of occurrence	The origin of the bacteria	Influence	Role
*Staphylococcus*	<10^5^ CFU ml^−1^	Acute prematurity	Caesarean section	Pathogenic	Unfolding mucin whose parts are used as food for the body. One of the first pathogens inhabiting the intestinal flora [Palmer et al., 2007]

Actinobacteria	10^4^ CFU ml^−1^	From the first years of life	With food	Beneficial	Coordination of the immune system Stimulation of gastrointestinal motility Production of bioactive compounds, including antibacterial, antifungal, and anthelmintic drugs as well as antitumor and antiviral drugs, for example, streptomycin and kanamycin [Kumar et al., 2010]

*Bacteroides*	10^10^ to 10^11^ per 1 g of the content	From the age of 3	With a decreasing amount of oxygen in the intestine There are obligatory anaerobes	Endogenous bacteria due to infection	Production of vitamins B7, B2, and C Inhibition of the growth of pathogens and harmful bacteria [Round et al., 2011] They stimulate the formation of iTreg [Round et al., 2011]

*Clostridium*	<10^5^ CFU ml^−1^	The largest number in the adulthood	With a decreasing amount of oxygen in the intestine There are obligatory anaerobes	Pathogenic	They stimulate the formation of Treg relevant in reducing the pathology dependent on Th2 cells in the mucous membranes of the respiratory system [Atarashi et al., 2011] [Josefowicz et al., 2012]

*Ruminococcus*	10^5^–10^6^ CFU ml^−1^	From the age of 3	Natural childbirth	Beneficial	Unfolding mucin, which helps the intestine absorb the fragments used as food for the body [Præsteng et al., 2013]

*Bifidobacteria*	10^10^ to 10^11^ CFU ml^−1^	From the first years of life if the child is breastfed	Mother's milk	Beneficial	They form a natural protective barrier against pathogens by producing bacteriocin and organic acids. Stimulation of gastrointestinal motility [Bottacini et al., 2014]

*Veillonella*	10^5^ CFU ml^−1^	From the first years of life	With food	Pathogenic	Coordination of the immune system Stimulation of gastrointestinal motility This increases the concentrations of IL-8, IL-6, IL-10, and TNF-*α* [van den Bogert et al., 2014]

*Prevotella*	10^6^-10^7^ CFU ml^−1^	From birth	Natural childbirth	Pathogenic	Production of vitamin B1 and folic acid Inhibiting the growth of pathogens and harmful bacteria [Koskey et al., 2014]

*Streptococcus*	<10^5^ CFU ml^−1^	From birth	The first organisms that colonize the intestine	Pathogenic	Stimulation of the immune system by the ability of adhesion to the bowel mucosa [Wang et al., 2014]

*Lactobacillus*	10^8^ to 10^10^ per 1 g of the content	From the first years of life if the child is breastfed	Natural childbirth	Beneficial	Create natural barriers to the growth of pathogenic bacteria by production of bacteriocin and organic acids [Bottacini et al., 2014]

*Escherichia coli*	>10^6^ CFU ml^−1^	The first few hours of life	With a decreasing amount of oxygen in the intestine There are obligatory anaerobes	Pathogenic	Production of vitamins B and K It produces fecal enzymes and synthesized carcinogens [Grudniak et al., 2015]

*Propionibacterium*	10^6^ CFU ml^−1^	From birth	Caesarean section	Pathogenic in immunocompromised individuals	Coordination of the immune system Stimulation of gastrointestinal motility Induced expression of proteins iNOS and COX-2 by ROS-dependent NF-kB and AP-1 activation of macrophages [Jahns et al., 2015]

iTreg: regulatory T cells; CFU: colony-forming unit; Th2: lymphocytes; IL: interleukin; TNF-*α*: tumor necrosis factor-alpha; iNOS: nitric oxide synthase; COX-2: induced cyclooxygenase; ROS: reactive oxygen species; NF-kB: transcription factor; AP-1: activator protein 1.

**Table 2:** Effect of gut microflora on the immune system in MS.

Authors	Materials	Models of diseases	Conclusions
Sriram et al. (1999)	17 patients with relapsing-remitting MS, 20 patients with progressive MS, and 27 patients with other neurological diseases (OND)	MS	CNS infections; *Chlamydia pneumoniae* is a common occurrence in patients with MS. Although *Chlamydia pneumoniae* may be a pathogenic factor of MS, it may simply be a secondary infection of damaged tissues of the CNS.

Becher et al. (2001)	Mice	EAE	CD40-CD154 interactions in the CNS are key determinants for the development and progression of the disease. No CD40 expression in cells of the CNS reduces the intensity and duration of myelin oligodendrocyte and glycoprotein-induced EAE and reduces the degree of infiltration of inflammatory cells into the CNS.

Oksenberg et al. (2008)	Mice	EAE	EAE is considered a model of autoimmune diseases, including MS.

Ezendam and van Loveren (2008); Ezendam et al. (2008)	Mice	EAE	Reduced symptoms in mice infected with *Lactobacillus casei* Shirota and *Bifidobacterium animalis*.

Yokote et al. (2008)	Mice	MS	Low-calorie diet alleviates the symptoms of MS.

Hawker et al. (2009)	Adults with primary progressive MS	MS	Rituximab monoclonal antibody, selective cell killing CD20, proved effective in reducing disease activity in relapsing-remitting MS.

Lavasani et al. (2009)	Mice with the strains of *Lactobacillus*	EAE	The administration of probiotic lactic acid has a positive effect on the autoimmune disease by the production of IL-10 and stimulation of Treg cells.

Barnett et al. (2009)	Patients with MS and other neurological diseases	MS	IgG disrupted myelin in MS.

Ochoa et al. (2010)	Mice after treatment with antibiotics and infected with *B. fragilis*	EAE	Antibiotic therapy can protect against EAE; a similar effect is observed for *B. fragilis* (commensal bacteria).

Farooqi et al. (2010)	Mice	EAE	EAE has similar features to inflammation, demyelination, axonal loss, and gliosis.

Lee et al. (2011)	Mice	EAE	SFB induce Th17 immune response.

Farrokhi et al. (2013)	Patients with and without MS concentration of lipid 624	MS	Lipid 624 (TLR2 ligand) occurs in lower concentrations in patients with MS [12].

Rumah et al. (2013)	Patients with MS and healthy individuals	MS	CSF obtained from two tissues; immunity to ETX is 10 times more frequent in individuals with MS compared to healthy subjects, indicating prior exposure to ETX in MS population.

Chiurchiù et al. (2013)	Healthy people and MS patients	MS	pDC from patients with MS and production of higher levels of interleukin-12 and interleukin-6, whereas pDC had lower levels of interferon-*α* compared to healthy subjects.

Tauschmann et al. (2013)	Healthy young people	Autoimmune disease	The imbalance between the bacteria and the intestinal immune system leading to overstimulation of the immune system.Treg cells have inhibitory effects on the cells in autoimmune diseases.

Reichelt et al. (2014)	Patients with MS	MS	The increase of IgA may be secondary to an increase in the intestinal absorption.

Miyake et al. (2015)	Patients with MS	MS	Patients with MS are characterized by moderate dysbiosis. The decrease in the percentage of several *Bacteroides*, including *B. stercoris*, *B. coprophilus*, and *B. coprocola*.

Nicol et al. (2015)	Mice	EAE	Reduction of the severity of symptoms after antibiotic treatment. Decrease in the amount of IFN-*γ*, MIP-1a, MIP-1 p, MCP-1, IL-17, and IL-6 and increase in the amount of IL-10 and IL 13.

EAE: experimental autoimmune encephalomyelitis; *B. fragilis*: *Bacteroides fragilis*; IL: interleukin; Treg: regulatory T cells; MS: multiple sclerosis; SFB: segmented filamentous bacteria; TLR2: Toll-like receptor 2; IgG: immunoglobulin G; Th17: T helper 17 cell; IFN-*γ*: interferon-gamma; MIP: macrophage inflammatory proteins; MCP-1: monocyte chemoattractant protein-1; IgA: immunoglobulin A; CSF: cerebrospinal fluid; *C. perfringens*: *Clostridium perfringens*; CD 40: cluster of differentiation 40; CD 154: cluster of differentiation 154; CNS: central nervous system; ETX: epsilon-toxin; pDC: proportion of days covered.

## Abbreviations

ATP: Adenosine triphosphate
CCL4: Chemokine ligand 4
CCL5: Chemokine ligand 5
CD4: Cluster of differentiation 4
CD8: Cluster of differentiation 8
CD39: Cluster of differentiation 39
CNS: Central nervous system
CRP: C-Reactive protein
DNA: Deoxyribonucleic acid
EAE: Experimental autoimmune encephalomyelitis
EBV: Epstein-Barr virus
ELISA: Enzyme-linked immunosorbent assay
FOXP3: Forkhead box P3
GALT: Gut-associated lymphoid tissue
G-CSF: Granulocyte colony-stimulating factor
GF: Germ-free
GPRS: Binding G protein coupled receptor
GWAS: Genome-wide association studies
HOMA-IR: Homeostasis Model Assessment of Insulin Resistance
HPA: Hypothalamic-pituitary-adrenal
IFN: Interferon
Ig: Immunoglobulin
IL: Interleukin
IgG2b: PE anti-mouse antibody
IP-10: Interferon-gamma-induced protein 10
LPS: Lipopolysaccharide
MCP-1: Monocyte chemoattractant protein-1
MHC: Major histocompatibility complex
MIP-1: Macrophage inflammatory protein 1
MS: Multiple sclerosis
p: Probability value
PSA: Polysaccharide A
ROS: Reactive oxygen species
RNS: Reactive nitrogen species
SCFA: Short-chain fatty acid
SLE: Systemic lupus erythematosus
SNP: Single-nucleotide polymorphism
Th1: Th cells
TLR: Toll-like receptors
TNF: Tumor necrosis factor
TNFR2: Tumor necrosis factor receptor 2
Treg: Regulatory T lymphocytes
UVR: Ultraviolet radiation.
